# The combined effect of systemic antibiotics and proton pump inhibitors on *Clostridioides difficile* infection and recurrence

**DOI:** 10.1093/jac/dkae012

**Published:** 2024-01-24

**Authors:** Nele Moreels, Annelies Boven, Oswaldo Gressani, Fredrik L Andersson, Erika Vlieghe, Steven Callens, Lars Engstrand, Johanna Simin, Nele Brusselaers

**Affiliations:** Department of Microbiology, Centre for Translational Microbiome Research, Tumour and Cell Biology, Karolinska Institutet, Stockholm, Sweden; I-BioStat, Data Science Institute, Hasselt University, Hasselt, Belgium; Department of Microbiology, Centre for Translational Microbiome Research, Tumour and Cell Biology, Karolinska Institutet, Stockholm, Sweden; Department of Family Medicine and Population Health, Global Health Institute, Antwerp University, Antwerp, Belgium; I-BioStat, Data Science Institute, Hasselt University, Hasselt, Belgium; Global Value & Access, Ferring Pharmaceuticals, Copenhagen, Denmark; Department of Family Medicine and Population Health, Global Health Institute, Antwerp University, Antwerp, Belgium; Department of Internal Medicine and Pediatrics, General Internal Medicine, Ghent University, Ghent, Belgium; Department of Microbiology, Centre for Translational Microbiome Research, Tumour and Cell Biology, Karolinska Institutet, Stockholm, Sweden; Department of Microbiology, Centre for Translational Microbiome Research, Tumour and Cell Biology, Karolinska Institutet, Stockholm, Sweden; Department of Microbiology, Centre for Translational Microbiome Research, Tumour and Cell Biology, Karolinska Institutet, Stockholm, Sweden; Department of Family Medicine and Population Health, Global Health Institute, Antwerp University, Antwerp, Belgium; Department of Public Health and Primary Care, Ghent University, Ghent, Belgium

## Abstract

**Background:**

Antibiotics and proton pump inhibitors (PPI) are recognized risk factors for acquisition and recurrence of *Clostridioides difficile* infection (CDI), yet combined effects remain unclear.

**Objectives:**

To assess the short- and long-term effects of antibiotics and PPIs on CDI risk and recurrence.

**Methods:**

Population-based study including all 43 152 patients diagnosed with CDI in Sweden (2006–2019), and 355 172 matched population controls without CDI. The impact of antibiotics and PPIs on CDI risk and recurrence was explored for recent (0–30 days) and preceding (31–180 days) use prior to their first CDI diagnosis, using multivariable conditional logistic regression presented as odds ratios (ORs) and 95% confidence interval, adjusted for demographics, comorbidities and other drugs.

**Results:**

Compared to controls, the combined effect of recent PPIs and antibiotics [OR_AB+PPI_ = 17.51 (17.48–17.53)] on CDI risk was stronger than the individual effects [OR_AB_ = 15.37 (14.83–15.93); OR_PPI_ = 2.65 (2.54–2.76)]. Results were less pronounced for exposure during the preceding months. Dose–response analyses showed increasing exposure correlated with CDI risk [recent use: OR_AB_ = 6.32 (6.15–6.49); OR_PPI_ = 1.65 (1.62–1.68) per prescription increase].

Compared to individuals without recurrence (rCDI), recent [OR_AB_ = 1.30 (1.23–1.38)] and preceding [OR_AB_ = 1.23 (1.16–1.31); OR_PPI_ = 1.12 (1.03–1.21)] use also affected the risk of recurrence yet without significant interaction between both. Recent macrolides/lincosamides/streptogramins; other antibacterials including nitroimidazole derivates; non-penicillin beta lactams and quinolones showed the strongest association with CDI risk and recurrence, particularly for recent use. PPI use, both recent and preceding, further increased the CDI risk associated with almost all antibiotic classes.

**Conclusion:**

Recent and less recent use of PPIs and systemic antibiotics was associated with an increased risk of CDI, particularly in combination.

## Introduction

*Clostridioides difficile* is responsible for one of the most feared healthcare-associated gastro-intestinal infections,^1–5^ and *C. difficile* infection (CDI) is a major global burden on healthcare facilities.^6–8^ Sweden has one of the highest reported incidences of CDI in Europe, with 60 cases per 100 000 inhabitants annually.^9^ Up to one-third of people with CDI relapse^10^ and the risk for recurrence increases with each episode.

*C. difficile* can be part of the normal healthy microbiome, but is also suspected to thrive in a dysbiotic or unhealthy gut.^11^ A significant factor affecting the microbiome composition is the use of prescribed and over-the-counter drugs.^12,13^ Therefore, it is unsurprising that drug use has been associated with CDI. Especially, previous exposure to antibiotics, in particular (third-generation) cephalosporins and clindamycin, is associated with an increased CDI risk.^14–18^ Furthermore, the use of gastric acid suppressants, including proton pump inhibitors (PPIs) and histamine-2 receptor antagonists (H2RA), as well as some non-steroidal anti-inflammatory drugs (NSAIDs), has been associated with CDI.^19–21^ Other unfavourable factors are older age (over 65 years), chronic comorbidities such as inflammatory bowel disease^22,23^ and hospital admission, especially at medical or general intensive care units, and long-term care facilities.^8,24,25^

As prescribed drug use is omnipresent and modifiable,^13^ it also provides an opportunity for CDI prevention. In Sweden, approximately 11% of adults use PPIs on a regular basis, and almost 20% of the total population and one-third of the elderly (80+ years) use antibiotics yearly.^13,26^ Although the impact of separate drug classes on the risk of (recurrent) CDI has been extensively investigated,^27,28^ potential drug interactions have rarely been explored despite the high prevalence of combination therapies.^29^ A US study including 241 cases with CDI concluded that PPI exposure was an independent risk factor for CDI, and suggested a statistical interaction with ‘low risk’ antibiotics.^30^ A more recent South Korean study, including 200 cases with CDI-associated diarrhoea on ‘high risk’ antibiotics, also concluded PPIs increase the CDI risk,^31^ yet both studies lacked power to look at individual antibiotic classes. Two meta-analyses have been recently published about PPI use as a risk factor for CDI: including 50 studies (OR = 1.26, 95% CI 1.12–1.39)^32^ and 67 studies (OR = 2.34, 95% CI 1.94–2.82).^19^ While both meta-analyses indicate a prominent increased risk of CDI among PPI users compared to non-users, all included studies are relatively small. The largest (US) study contained 5967 cases of CDI,^33^ and the larger meta-analysis incorporated a total of 17 317 cases.^19^ Consequently, for interactions between different drug classes, neither duration nor (cumulative) dosage of drugs have been studied sufficiently.

To better understand the association between prescribed drug use and the risk of (recurrent) CDI, it is important to investigate large cohorts to explore the potential interaction effects between different prescribed drugs. Among the commonly prescribed drug classes already established as risk factors for CDI, PPIs and antibiotics seem to be the most important disruptors of the microbiome at a population level.^12,34,35^ It is suggested that the adult microbiome mostly recovers within 1.5 months after antibiotic exposure, but some common species remained absent up to 180 days.^36^ Yet, maintenance use e.g. by PPIs, may even have more prominent and/or lasting effects on the microbiome than antibiotics.^12,34^ Therefore, differences in the short- and long-term effects of prescription drugs need exploration.

In this study, the association between antibiotics and PPIs, and their combined effects, and the risk and recurrence of CDI is explored using the nationwide and population-based Swedish health registries.

## Materials and methods

### Study design and data

**Figure 1.**
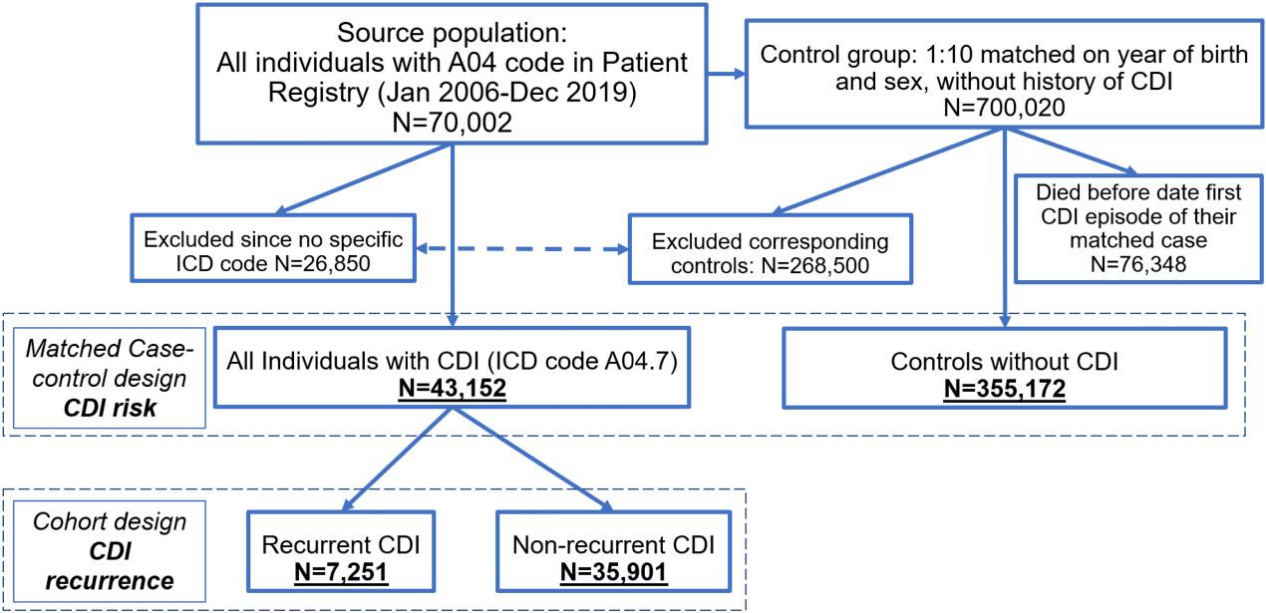
Flowchart describing the selection of participants in the case-control design to assess *CDI* risk, and the cohort design to assess CDI recurrence. This figure appears in colour in the online version of *JAC* and in black and white in the print version of *JAC*.

This nationwide population-based study included all individuals with a recorded CDI diagnosis (defined by the ICD-10 code A04.7) in Sweden between 1 January 2006 and 31 December 2019 (*N* = 43 152), individually matched to 10 controls (Supplementary methods, available as Supplementary data at *JAC* Online).^37^ Our large CDI database has been constructed to assess risk factors for CDI, prognosis and healthcare burden of CDI. In this database, all cases with the slightly broader ICD-10 code of A04 (instead of A04.7) were individually matched by the National Board of Health and Welfare, to up to 10 controls based on year of birth and sex (Figure 1). Controls were individuals who had received at least one dispensed drug prescription between 2006 and 2019, as determined by the Swedish Prescribed Drug Registry (outpatient care drug use based on Anatomical Therapeutic Chemical (ATC) codes, from July 2005). Indications of use are not recorded in the Prescribed Drug Registry. Controls were not allowed to have a history of CDI, defined as having no CDI episodes since 1997 (when ICD-10 was introduced). Note that 76 348 controls died before their proxy date (i.e. the date of the first CDI episode of their corresponding case), and they were consequently removed from the final dataset (*N* = 355 172). As matching was based on year of birth, we used the age of the CDI cases for their matched controls.

We use a matched case-control design to assess the risk factors for CDI (using the entire database including all CDI cases and their controls); and a longitudinal cohort design to assess the risk of recurrence among those individuals with a CDI episode (only including those with CDI). As previously described, 91.6% of all cases with CDI were considered hospital-acquired, 7.2% community-acquired and 1.2% had an unclear origin.^38^

### Exposures

Based on the ATC classification system, systemic antibiotic use (J01) consists of 11 classes: tetracyclines (J01A), amphenicols (J01B), β-lactam antibacterials, penicillins (J01C), other β-lactam antibacterials (J01D), sulphonamides and trimethoprim (J01E), macrolides, lincosamides and streptogramins (J01F), aminoglycoside antibacterials (J01G), quinolone antibacterials (J01M), combinations of antibacterials (J01R) and other antibacterials including among others imidazole derivates and nitrofurans (J01X). J01X was merged with nitroimidazole derivates (P01AB) as these also include imidazole derivates. PPIs were defined by ATC-code A02BC.

Short- and long-term effects were investigated by exploring the impact of these outpatient prescription drugs within two time periods—‘recent’ (0–30 days) and ‘preceding’ (31–180 days)—prior to the first CDI diagnosis during the study period. Exposure was defined as at least one prescription (i.e. one package) within the study period. The utilization units for drugs were the number of prescriptions, and estimated duration of treatment based on the DDDs in dispensed packages, given that one DDD corresponds to 1 day of treatment for the standard indication in adults.^39–42^

### Outcomes

Two models were used: one to assess CDI risk, comparing CDI cases to their matched controls; and one to assess CDI recurrence (within 8 weeks after the initial episode), comparing recurrent CDI versus non-recurrent CDI. Recurrent CDI was defined as a new episode within 8 weeks from the initial CDI diagnosis.^43^

Only the first CDI episode recorded during the study period was included to determine CDI risk. For recurrence, all individuals with at least one recurrence were compared to those with CDI but without recurrence during the study period.

### Covariates

Other potential risk factors included patient characteristics (sex, age at diagnosis, region of birth), and chronic comorbidities (Table S1). History of CDI was only included in the recurrence models, as none of the controls could have had CDI history (to avoid long-lasting health/microbiome effects related to CDI or CDI treatment in our control group). Exposure to other prescription drugs [H2-receptor antagonists (H2RA, ATC and A02BA), aspirin (ATC, B01AC06 and N02BA) and NSAIDs (ATC, M01A)] was also considered because these drugs may also affect the microbiome (defined as at least one prescription during the last 6 months).^13,35^

### Statistical analysis

Multivariable conditional logistic regression was used to assess the association between prescribed drug use and CDI risk, distinguishing between recent and preceding use, and compared to matched controls without CDI. These models accounted for the matching procedure,^44^ and were presented as ORs with 95% CIs. The full model included all main effects and the interaction terms of interest between the antibiotic use (overall and by ATC subclass) and PPIs, and controlled for region of birth, comorbidities and other drug use.

In addition, multivariable logistic regression was used to assess the risk of CDI recurrence associated with antibiotic exposure and PPI use compared to those without recurrence in two time periods befire the initial CDI diagnosis, while adjusting for age (continuous variable), sex, number of comorbidities (continuous variable), other drug use and history of CDI, presented as adjusted ORs and 95% CIs.

To investigate the dose–response association, the number of prescriptions and estimated number of exposed days (based on the defined daily dosage per package) were assessed in relation to CDI risk and recurrence.^40–42^

## Results

The dataset included the 43 152 individuals with CDI and 355 172 matched controls (Table 1, Figure 1). Of the CDI group, 17% had a recurrence within 8 weeks. Antibiotics and PPIs were markedly more common among those with CDI compared to the controls. Overall, 63% of those with CDI were at some point exposed to antibiotics (6 months before CDI) compared to 16% of the controls, and 39% (CDI) and 14% (controls) were exposed to PPIs (Table 1). Antibiotic (69% versus 62%) and PPI (41% versus 38%) consumption was slightly higher among those with recurrence compared to those without.

**Table 1.** Descriptive characteristics and recent drug exposure of all individuals with one or multiple episodes of CDI in Sweden (2006–2019), and their matched controls

Characteristic		Non-recurrent CDI *N* (%)	Recurrent CDI *N* (%)	Total CDI *N* (%)	Controls *N* (%)
Total		35 901 (83.20)	7251 (16.80)	43 152 (100.00)	355 172 (89.17)
Systemic antibiotic use	Any	22 205 (61.85)	5021 (69.25)	27 226 (63.09)	55 308 (15.57)
	0–30 days	4099 (11.42)	989 (13.64)	5088 (11.79)	7028 (1.98)
	31–180 days	10 167 (28.31)	2146 (29.60)	12 313 (28.53)	42 084 (11.85)
	Both	7939 (22.12)	1886 (26.01)	9825 (22.77)	6196 (1.74)
	None	13 696 (38.15)	2230 (30.75)	15 926 (36.91)	299 864 (84.43)
PPI use	Any	13 746 (38.29)	2982 (41.13)	16 728 (38.77)	49 205 (13.85)
	0–30 days	1449 (4.04)	289 (3.99)	1738 (4.03)	2798 (0.98)
	31–180 days	6980 (19.44)	1541 (21.25)	7175 (16.63)	29 990 (8.44)
	Both	5317 (14.81)	1152 (15.89)	6469 (14.99)	16 417 (4.62)
	None	22 155 (61.71)	4269 (58.87)	26 424 (61.23)	305 967 (86.15)
Sex	Male	16 636 (46.34)	3145 (43.37)	19 781 (45.84)	159 897 (45.02)
	Female	19 265 (53.66)	4106 (56.63)	23 371 (54.16)	195 275 (54.98)
Age at first CDI diagnosis^a^, years	0–18	1301 (3.62)	281 (3.88)	1582 (3.67)	15 814 (4.45)
	19–40	2081 (5.79)	348 (4.80)	2429 (5.63)	24 238 (6.82)
	41–64	5801 (16.16)	1072 (14.78)	6873 (15.93)	67 493 (19.00)
	≥65	26 718 (74.42)	5550 (76.54)	32 268 (74.78)	247 627 (69.72)
Region of birth	Nordic	33 337 (92.86)	6775 (93.44)	40 112 (92.95)	238 711 (67.21)
	Non-Nordic	1907 (5.31)	354 (4.88)	2261 (5.24)	19 887 (5.60)
	Missing	657 (1.83)	122 (1.68)	779 (1.81)	96 574 (27.19)
Chronic comorbidities	Yes	30 723 (85.58)	6 303 (86.93)	38 681 (89.64)	218 272 (61.46)
	No	5178 (14.42)	948 (13.07)	4471 (10.36)	136 900 (38.54)
History of CDI (1997–2005)	Yes	362 (1.01)	140 (1.93)	502 (1.16)	0 (0.00)
	No	35 539 (98.99)	7111 (98.07)	42 650 (98.84)	355 172 (100.00)
Other drug use (NSAIDs, aspirin or H2RA)	0–30 days	812 (2.26)	183 (2.52)	995 (2.31)	4521 (1.27)
	31–180 days	8407 (23.41)	1742 (24.02)	10 149 (23.52)	65 799 (18.53)
	Both	5108 (14.23)	1023 (14.11)	6131 (14.21)	33 945 (9.56)
	None	21 574 (60.09)	4303 (59.34)	25 877 (59.97)	250 907 (70.64)

H2RA, histamine-2 receptor antagonists. History of CDI was defined as at least one recorded CDI diagnosis since 1997; population controls were 1:10 matched based on year of birth and sex.

^a^Age for controls corresponds to the age of their matched case at the time of their first recorded CDI episode.

Penicillins (J01C), other bacterials (J01X and P01AB) and quinolones (J01M) were most common among CDI cases and controls; both for recent use and preceding use (Table S2). As expected, those with CDI had more often chronic comorbidities (86%) compared to the controls (61%).

### Antibiotics and PPI versus CDI risk

**Figure 2.**
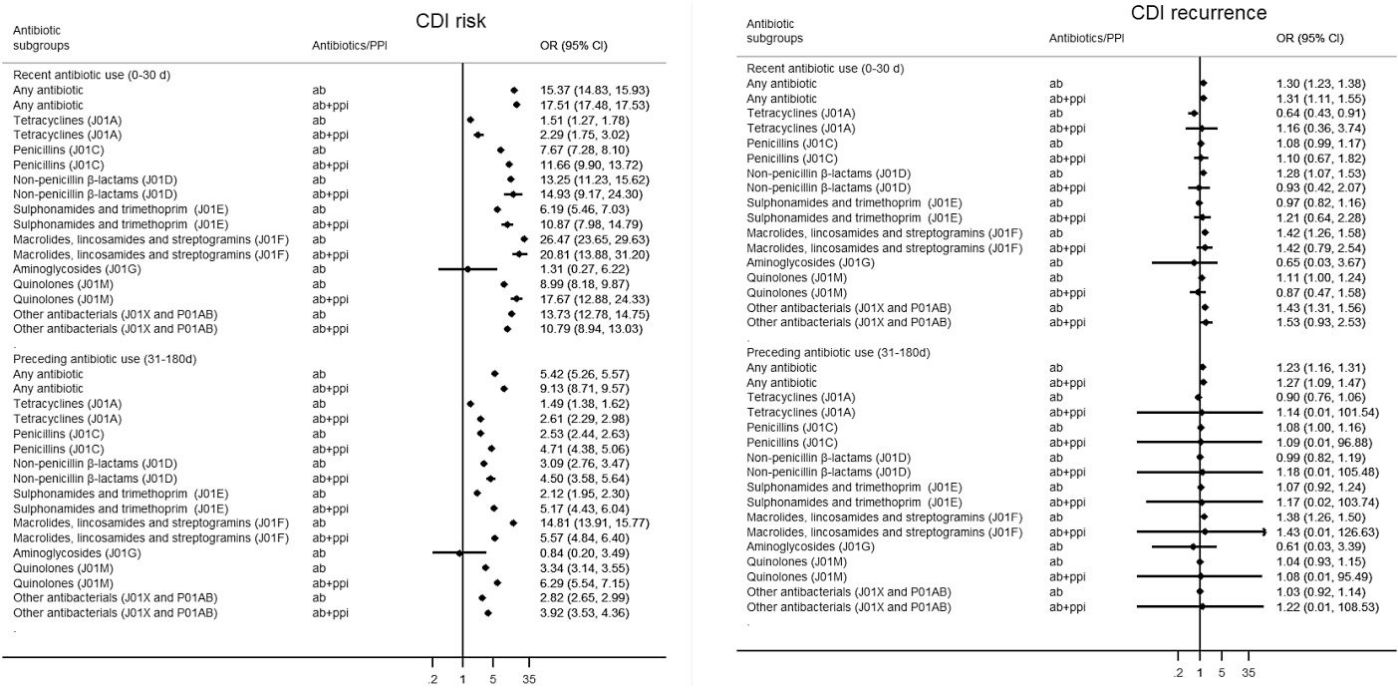
Effect of (combined) use of antibiotics and PPI on (a) the risk of CDI and (b) CDI recurrence, expressed as OR. All models were adjusted for region of birth, chronic comorbidity score (continuous variable), inflammatory bowel disease, haematological diseases, aspirin, NSAID use and H2-receptor antagonist use. The recurrence model was additionally adjusted for sex and age (continuously).

Both antibiotics and PPIs were associated with increased odds of CDI, with an even stronger effect when combined (Figure 2, Table S3). If recently exposed to both antibiotics and PPIs, the odds of CDI were 17.51 (95% CI 17.48–17.53) higher than among non-users, compared to OR = 15.37 (95% CI 14.83–15.93) for only antibiotics and OR = 2.65 (95% CI 2.54–2.76) for PPIs. The interaction term of OR = 0.69 (95% CI 0.67–0.71) suggests, however, that the increased risk related to PPIs is lower for a patient on antibiotics than for a patient who does not take antibiotics.

For preceding use, less pronounced yet still clearly increased effects were seen with OR = 9.13 (95% CI 8.71–9.57) for combined effect, OR = 5.42 (95% CI 5.26–5.57) for antibiotics only and OR = 2.08 (95% CI 2.01–2.15) for PPI alone.

An interaction between PPI and antibiotics was seen for recent and preceding use of all antibiotic subclasses, with the combined effect being more extreme for all subclasses except for macrolides and others (J01F) and other antibacterials (J01X/P01AB). Those two subclasses were responsible for the strongest effect (largest odds ratios) on CDI independent of PPI exposure, with OR = 26.47 (95% CI 23.65–29.63) for J01F and OR = 13.73 (95% CI 12.78–14.75) for J01X/P01AB, and OR = 20.81 (95% CI 13.88–31.20) for J01F and OR = 10.79 (95% CI 8.94–13.03) for J01X/P01AB when combined with PPI. The strongest interaction with PPIs was seen for quinolones (J01M), where the odds for CDI doubled from OR = 8.99 (95% CI 8.18–9.87) to OR = 17.67 (95% CI 12.88–24.33) if PPIs were combined with antibiotics.

Interactions between antibiotic subclasses and PPIs were also found for preceding use; with the strongest combined effect for quinolones (J01M) with OR = 6.29 (95% CI 5.54–7.15), while quinolones without PPIs resulted in OR = 3.34 (95% CI 3.14–3.55).

### Antibiotics and PPI versus CDI recurrence

Recent PPI use was not associated with CDI recurrence (OR = 1.03, 95% CI 0.94–1.12) (Figure 2, Table S3). Preceding PPI use, however, was associated with an increased risk of CDI (OR = 1.12, 95% CI 1.03–1.21). Recent antibiotic use did result in slightly higher odds of CDI recurrence (OR = 1.30, 95% CI 1.23–1.38) than older use (OR = 1.23, 95% CI 1.16–1.31), and the risk of recurrence barely changed if combined with PPIs. There was no interaction between PPIs and antibiotic subclasses regarding CDI recurrence, except for quinolones, where the risk slightly decreased when combined with PPIs (OR = 1.11, 95% CI 1.00–1.24 to OR = 0.87, 95% CI 0.47–1.58).

### Dose–response association

Regarding CDI risk, each recent prescription of PPIs and antibiotics resulted in increased odds of CDI, with OR = 1.65 (95% CI 1.62–1.68) for PPIs and OR = 6.32 (95% CI 6.15–6.49) for antibiotics (Table 2). Each prescription during the preceding months was also associated with increased CDI odds, with OR = 1.10 (95% CI 1.09–1.10) for PPIs and OR = 1.72 (95% CI 1.72–1.74) for antibiotics. When expressed as the number of days, the effect per 10 days was stronger for antibiotics than for PPIs, both for recent and preceding use.

**Table 2.** Dose–response analyses for each prescription and day exposed to antibiotics or PPI, and the risk of CDI and recurrence, expressed as OR and 95% CI

	First episode (compared to controls)		Recurrence (compared to no recurrence)	
	Recent (0–30 days)	Preceding 5 months (31–180 days)	Recent (0–30 days)	Preceding 5 months (31–180 days)
Number of prescriptions (per prescription)				
PPI	1.65 [1.62; 1.68]^a^	1.10 [1.09; 1.10]^a^	1.01 [0.97; 1.05]	1.00 [0.99; 1.01]
Systemic antibiotics (AB)	6.32 [6.15; 6.49]^a^	1.72 [1.71; 1.74]^a^	1.15 [1.11; 1.18]^a^	1.03 [1.02; 1.05]^a^
Interaction term PPI and AB	0.69 [0.67; 0.71]^b^	0.97 [0.97; 0.97]^b^	0.99 [0.96; 1.01]	1.00 [1.00; 1.00]
Number of days exposed (per 10 days)				
PPI	1.010 [1.010; 1.011]^a^	1.002 [1.002; 1.002]^a^	1.000 [0.999; 1.001]	1.000 [1.000; 1.000]
Systemic antibiotics (AB)	1.071 [1.068; 1.073]^a^	1.080 [1.076; 1.083]^a^	1.046 [1.026; 1.065]^a^	1.005 [1.000; 1.011]
Interaction term PPI and AB	0.997 [0.996; 0.997]^b^	1.000 [1.000; 1.000]^b^	1.000 [1.000; 1.000]	1.000 [1.000; 1.000]

^a^Significant association.

^b^Significant interaction between antibiotic and PPI. All models were adjusted for region of birth, chronic comorbidity score (continuous variable), inflammatory bowel disease, haematological diseases, aspirin, NSAID use and H2-receptor antagonist use. The recurrence model was additionally adjusted for sex and age (continuously).

Compared to those without recurrence, the number of PPI prescriptions was not associated with CDI recurrence, although the number of antibiotic prescriptions did increase the risk of recurrence (OR = 1.15, 95% CI 1.11–1.18 per prescription).

### Other potential predictors of CDI risk and recurrence

As expected, the Charlson comorbidity score was associated with increased odds of CDI, with each additional comorbidity resulting in 30% higher odds of CDI (OR = 1.30, 95% CI 1.29–1.31) (Table S4). Inflammatory bowel disease and haematological diseases also contributed to higher odds of CDI, with OR = 4.84 (95% CI 4.50–5.19) and OR = 3.10 (95% CI 3.02–3.19) respectively. Aspirin use seemed protective (OR = 0.86, 95% CI 0.83–0.90), while both H2-receptor antagonists (OR = 1.72, 95% CI 1.41–2.11) and NSAIDs (OR = 1.24, 95% CI 1.16–1.33) appeared to increase the odds of CDI.

Recurrence was more common in women than men (OR = 1.14, 95% CI 1.08–1.20) and comorbidities were associated with higher ratios (OR = 1.03, 95% CI 1.02–1.04 per additional comorbidity). History of CDI also seemed an important predictor for recurrence (OR = 1.84, 95% CI 1.50–2.23), but other drug use did not affect recurrence risk.

## Discussion

This large Swedish population-based study showed that recent and preceding outpatient use of all antibiotic classes, except for the rarely used aminoglycosides (J01G), was associated with a significantly increased risk of CDI with the J01F class of macrolides, lincosamides and streptogramins showing the largest increase (OR = 26). There was an interaction with PPIs, particularly with quinolones (J01M), for which the OR of CDI went up from 9 to 18 compared to population controls. Compared to those with CDI yet without recurrence, antibiotic use prior to the first episode seemed predictive for only some antibiotic classes with limited interaction with PPIs.

Our results support previous evidence for clindamycin and other J01F-type antibiotics and J01D beta-lactam antibacterials to be among the most prominent risk factors for CDI.^14,15,17,18,27^ A recent large case-control study from the USA (*N* = 159 404 CDI cases)^27^ also showed some very large effect sizes, with their strongest association between clindamycin (J01FF, OR = 25.4, 95% CI 24.1–16.0), very close to our OR = 26.5 (95% CI 23.7–29.6) for the J01F class. As expected, a recent exposure to antibiotics has a higher impact compared to preceding usage, yet for PPIs this difference was less pronounced. A potential reason is the microbiome still being disrupted and unbalanced—and hence more susceptible after antibiotic exposure,^36^ while PPIs are often used over prolonged periods of time, hindering microbiome restoration and facilitating a continued state of dysbiosis. For recurrence, we only looked at exposure prior to the first CDI episode, since clinically, it would be useful to predict who is most likely to have recurrence from the start, as this may alter follow-up and treatment decisions. Follow-up may be more frequent for individuals with a higher risk for recurrence, or duration and dosing of antibiotics may be adapted. Our findings suggests that individuals who acquired CDI after exposure to macrolides, lincosamides or streptogramins (J01F) are those most likely to experience a recurrence (OR = 1.42), with a similar risk for those on other bacterials (J01X/P01AB; OR = 1.43), and also an important risk increase for non-penicillin beta lactams (OR = 1.28). However, the odds of recurrence did not increase dramatically when combined with PPIs. The finding that preceding use of PPIs was significantly associated with recurrence, but not recent use may reflect more chronic use in the preceding group, or could be a chance finding.

Strengths of this study are the large population, valid high-quality and nationwide data sources, and use of matched population controls, limiting the risk of selection and information bias. We also adjusted all results, where feasible, for chronic comorbidities, sex, age, other drug use, history of CDI and region of origin, a proxy for ethnicity, diet and lifestyle (which are not collected in the Swedish nationwide registries). By including up to 10 controls per individual, the effect of residual confounders should also be limited. In particular, the Swedish Prescribed Drug Registry is extremely valuable from a global perspective, enabling investigation of large populations over extended periods of time, and a detailed assessment by antibiotic class.^45,46^ Our results should be generalizable to other populations with similar patterns of antibiotic and PPI consumptions, and similar prevalence of CDI, although different strains and antimicrobial resistance may affect these associations. Our prevalence of CDI recurrence (17% within 8 weeks, 29% within 20 weeks) was comparable to described literature.^10^ The risk of misclassification of exposure to antibiotics should be limited as these are only available on prescription, yet we only caught outpatient use. PPIs, however, are available over-the-counter, but only in small packages at a higher price so we assume regular users are more likely to use prescribed PPIs. Compliance with the prescribed treatment cannot be assessed, although all packages were dispensed. Actual duration of use may be lower for antibiotics as it was based on the DDD per package and not the actual/prescribed duration, which was not available for analyses.

Although the Swedish Patient Registry is regarded as a valid source for multiple diagnoses,^47^ our biggest limitation is the limited validity of our CDI diagnosis, based on reporting in the Swedish registries, without available clinical data on CDI severity. Reporting CDI is not mandatory in Sweden, only when there is a severe outbreak, and the incidence might therefore be underestimated and biased towards more severe cases, as also described in an earlier French validation study reporting low sensitivity but high specificity of ICD-10 based CDI.^48^ Some controls may have experienced unrecorded CDI as well. With 3082 cases over a 14-year period in a population of approximately 10 million, the CDI incidence was approximately 3.1 cases per 10 000 person years. Confounding by indication may play a role, with antibiotic/PPI users being less healthy than those not exposed, but indication of drug use is not registered in the Drug Registry, with only specialist-outpatient diagnoses and hospital discharge diagnoses being available. Despite matching on age, 90% of the CDI group had chronic comorbidities and were therefore more likely to be exposed to prescription drugs; with only 62% of the controls presenting with chronic comorbidities. Our population-based design did not incorporate matching on hospitalization status or frequency, which are also closely correlated to comorbidities. As mentioned before, we only assessed drug exposure prior the CDI episode, but we acknowledge that the recurrence risk may be affected by the (antimicrobial) CDI treatment, of which we have incomplete information as in-patient drugs are not collected in the Drug Registry. In Sweden the choice of treatment depends on the severity of the infection and the estimated recurrence risk, with an estimated 25% expected to recover without any treatment within 3–4 days.^49–51^ For moderate infections, metronidazole is recommended, whereas vancomycin is preferred for intermediate infections with therapy failure or severe infections.^49–51^ Metronidazole combined with vancomycin is recommended for fulminant colitis.^49–51^ For repeated recurrent infections there is currently no available treatment policy. Faecal microbiota transplantation is an alternative, yet no nationwide stool banks are yet available.^52–54^ Vancomycin with decreasing intermediate dosage, for 6 weeks, may be considered in highly resistant cases.^49^ We opted for (conditional) logistic regression models for CDI risk and recurrence to obtain OR and 95% CI in all models and facilitate interpretation, and also because follow-up time would have to be restricted to 8 weeks to adhere to the standard definition of recurrence. Nevertheless, our previous work did show a non-negligible mortality with 9.2% dying within 30 days,^38^ suggesting death is an important competing risk that should be explored in further research.

Except for aminoglycosides (J01G), all other antibiotic classes showed significantly increased odds of CDI. A recent systematic review including 78 studies summarized current knowledge regarding the effects of antibiotics on the microbiome.^55^ Nevertheless, findings were often based on a limited number of relatively small studies that often looked at genus level and not specific species, as in a similar overview paper on PPIs.^56^ This systematic review has highlighted the consistent changes of quinolone and metronidazole on the microbiome, two classes also showing high CDI risks in the present study.

Regarding the microbiome-susceptibility to CDI, pointing to a single genus or species or even one anatomical niche is too simplistic, as many variables are influencing the different microbiota compositions in the body. Previous studies described that nitrofurans and tetracyclines may reduce the number of *Clostridia* class.^55,56^ PPIs have been associated with an important disruption of the gut microbiome, on a population level potentially bigger than antibiotics^12,34,57–63^ as PPIs are often (inappropriately) used as maintenance therapy (in 10%–30% of adults), whereas antibiotics are usually administered over shorter periods of time.^59–62,64^ PPIs were associated in particular with a decreased abundance of *Ruminococcocea* and increased Enterobacteriaceae, *Enterococcoceae* and *Lactobacillaceae*, creating a pro-inflammatory environment.^35,56,65^ An increase in the *Clostridiaceae* family was related to PPI use in the oesophagus and small intestine but not in the colon.^56^ Combined administration of different antibiotic classes may also result in interactions and potential bigger disruptions of the microbiome, as the spectrum of antibacterial activity broadens.^55^ H2Ra are clearly less potent than PPIs,^66^ yet also less frequently used; in Sweden, maintenance therapy with PPIs is almost 40 times more frequent than by H2RA.^26^ This may partially explain why the effect on the microbiome is less pronounced and fewer negative health effects have been described for H2RA than PPIs in association studies.^66–68^ In our present study, we did, however, find an effect of H2Ra on CDI risk (OR 1.72, 95% CI 1.41; 2.11, Table S4), although less pronounced than PPIs.

The findings of this study add more weight to the evidence on the role of prescription drugs and the risk of CDI and reveals the even stronger combined effect. This article only investigates one piece of the puzzle regarding drug interactions in relation to CDI risk, especially among this older age group most at risk for CDI, where polypharmacy is rampant. Our analyses also looked at broader exposure periods and broad drug classes (according to the large ATC categories), while different mechanisms of action and effects could be expected if we would subdivide into even smaller groups, e.g. by (an)aerobic targets. A more in-depth analysis stratifying drug-related risks by comorbidity and other characteristics may also provide useful clinical insights, as well as assessment of the effects of other drug groups.

Our findings stress the need to reconsider the risk-benefit of both antibiotics and PPIs, which are both still over-prescribed.^64,69^ Furthermore, since antibiotics are also used to treat CDI, assessing the impact of these dispensed prescriptions on the odds of a recurrent episode is important to investigate, as well as the long-term efficacy and safety of the treatment.

In conclusion, exposure to systemic antibiotics and the use of PPIs were associated with CDI risk and recurrence, especially when combined.

## Supplementary Material

dkae012_Supplementary_Data

## Data Availability

The datasets generated and analysed are not publicly available due to restrictions from the National Board of Health and Welfare, the owners of the data. The data are available from the corresponding author (N.B.) on reasonable request after required approvals from the national Ethics Committee and National Board of Health and Welfare are obtained.

## Supplementary data

Tables S1 to S4 are available as Supplementary data at *JAC* Online.
