# Supplementary material for: The combined effect of systemic antibiotics and proton pump inhibitors on Clostridioides difficile infection and recurrence
Source: J Antimicrob Chemother. 2024 Jan 24;79(3):608–16. doi: 10.1093/jac/dkae012 (PMC10904719; doi:10.1093/jac/dkae012)
Supplement: dkae012_Supplementary_Data [file dkae012_supplementary_data.docx]

**Supplementary methods**

Individual patient data were acquired from the following high-quality nationwide Swedish registries: the Prescribed Drug Registry,[^1^](#_ENREF_1) the Patient Registry (in- and outpatient),[^2^](#_ENREF_2) and the Cancer Registry,[^3^](#_ENREF_3) and linked by their unique personal identifier assigned to each Swedish resident. The Prescribed Drug Registry, which includes all Swedish residents who received at least one prescription drug during 2006-2019. This source encloses almost the entire Swedish population as, annually, more than 65% use prescription drugs, and even 90% of those above 60 years.[^4^](#_ENREF_4) Chronic comorbidities are represented by the Charlson comorbidity index,[^5^](#_ENREF_5)^,^ [^6^](#_ENREF_6) inflammatory bowel disease and haematological diseases), as retrieved from the National Patient Registry.

As a large proportion of the controls (27%) lacked information on region of birth (Nordic or Non-Nordic), potentially because this information got lost during the matching, we presumed that the individuals with missing information were Nordic-born (to be able to conduct complete-case analyses without losing individuals). This resulted in a similar distribution as among individuals with CDI.

The ORs for the combined exposure to antibiotics and PPIs (OR_AB+PPI_) were calculated multiplying OR_AB_ * OR_PPI_ * OR_interactionterm_; and the confidence intervals were obtained by the following formulae:[^7^](#_ENREF_7) CI_AB+PPI_= exp(logOR_AB+PPI_ ±1.96*SE_AB+PPI_); SE_AB+PPI_= sqrt((J+1/J+2)/n_AB+PPI_ + (SEintercept)^2); and J= OR_AB_ * OR_PPI_ * OR_intercept_ * OR_interactionterm_

As the conditional logistic regression model to calculate CDI risk does not provide an intercept, we used OR_intercept_=0. As age and sex were used as matching variables in this case-control design, their effect on the risk of CDI could not be assessed but was assessed for the risk of recurrence. Significance levels are fixed at 5%.

**Table S1**. International Classification of Disease (ICD-10) codes to define Chronic comorbidities, including those included in the Charlson Comorbidity Index score, based on the Swedish Patient Registry.

| **Comorbidity** | **Charlson Comorbidity index weight** | **Corresponding ICD-10 codes** |
| --- | --- | --- |
| Myocardial infarction | 1 | I21-I23, I241, I252 |
| Congestive heart failure | 1 | I110, I130, I132-255, I420, I425-I439, I50, K761 |
| Peripheral vascular disease | 1 | A520, I70-I72, I731, I738, I739, I771, I778, I790, K441 |
| Cerebrovascular disease | 1 | G45-G46, I60–I69, H340 |
| Dementia | 1 | F00–F03, F051, G30, G311 |
| Chronic pulmonary disease | 1 | I270, I278, I279, J40-J47, J60-J67, J684, J701, J703 |
| Connective tissue disease | 1 | M05-M06, M08-09, M315, M32–M34, M351, M353 |
| Ulcer | 1 | K25-K28 |
| Mild (chronic) liver disease | 1 | B18, K700-K704, K709, K710, K713-K719, K73-K74, K760 |
| Diabetes mellitus without end-organ damage | 1 | E109, E119 |
| Any malignancy*, including metastatic cancer, leukaemia, and lymphoma | 2 | C00-C97, ** omitting non-melanoma skin cancer C43.0-9 and C87 (which is not used in Sweden)*[*^6^*](#_ENREF_6) |
| Diabetes mellitus with end-organ damage | 2 | E129, E139, E149, E100-E108, E110-E118, E120-E128, E130-E138, E140-E148 |
| Hemiplegia or paraplegia | 2 | G041, G114, G801, G802, G810-G82 |
| Moderate to severe chronic renal disease | 2 | I120, I131, I132, N03-N05, N18-N19, N25-N26, Z49 |
| Moderate or severe liver disease | 3 | I810-I820, I85, I864, K704, K711, K712, K72, K762, K763, K765-K767 |
| AIDS/HIV | 6 | B200–B248 |
| Irritable bowel disease | - | K500-K501, K508, K510, K512, K515, K518, K513, K523 |
| Haematological disease | - | D50-D89 |

**Table S2. Number of individuals exposed to the different antibiotic types and proton pump inhibitors (PPIs) with and without *Clostridioides difficile* infection (CDI), by recurrence (r) status.**

|  | **Recent exposure (0 – 30 days)** | | | | | **Exposure during preceding 5 months (31 – 180 days)** | | | | |
| --- | --- | --- | --- | --- | --- | --- | --- | --- | --- | --- |
|  | All CDI | non-rCDI | rCDI | Controls | Total | All CDI | non-rCDI | rCDI | Controls | Total |
| Total | 43,152 | 35,901 | 7,251 | 355,172 | 398,324 | 43,152 | 35,901 | 7,251 | 355,172 | 398,324 |
| Any PPI use | 8,206 | 6,765 | 1,441 | 18,688 | 26,894 | 14,988 | 12,295 | 2,693 | 46,407 | 61,395 |
| No PPI | 34,946 | 29,136 | 5,810 | 336,484 | 371,430 | 28,164 | 23,606 | 4,558 | 308,765 | 336,929 |
| Any systemic antibiotics use | 14,913 | 12,038 | 2,875 | 11,131 | 26,044 | 22,136 | 18,104 | 4,032 | 48,280 | 70,416 |
| *Tetracyclines (J01A)* | 341 | 296 | 45 | 1317 | 1,658 | 2,030 | 1,688 | 342 | 7,373 | 9,403 |
| *Penicillins (J01C)* | 5935 | 4868 | 1067 | 5309 | 11,244 | 12,364 | 10,139 | 2,225 | 28,633 | 40,997 |
| *Non-penicillin β-lactams (J01D)* | 991 | 791 | 200 | 370 | 1,361 | 1,427 | 1,169 | 258 | 1,707 | 3,134 |
| *Sulphonamides and trimethoprim (J01E)* | 1304 | 1074 | 230 | 911 | 2,215 | 2,772 | 2,266 | 506 | 4,110 | 6,882 |
| *Macrolides, lincosamides and streptogramins (J01F)* | 2391 | 1871 | 520 | 684 | 3,075 | 6,496 | 5,138 | 1,358 | 3,267 | 9,763 |
| *Aminoglycosides (J01G)* | 8 | 7 | 1 | 6 | 14 | 21 | 18 | 3 | 5 | 26 |
| *Quinolones (J01M)* | 2842 | 2312 | 530 | 1272 | 4,114 | 5,544 | 4,553 | 991 | 6,418 | 11,962 |
| *Other antibacterials (J01X and P01AB)* | 4432 | 973 | 3459 | 2279 | 6,711 | 5,045 | 933 | 4,112 | 7,962 | 13,007 |
| No antibiotic use | 28,239 | 23,863 | 4,376 | 344,041 | 372,280 | 21,016 | 17,797 | 3,219 | 306,892 | 327,908 |
| Combined systemic antibiotic and PPI use | 3,161 | 2,538 | 623 | 1,656 | 4,817 | 8,952 | 7,271 | 1,681 | 10,662 | 19,614 |
| *Tetracyclines (J01A)* | 68 | 55 | 13 | 175 | 243 | 454 | 372 | 82 | 672 | 1,126 |
| *Penicillins (J01C)* | 1,289 | 1,052 | 237 | 701 | 1,990 | 2,586 | 2,113 | 473 | 2,477 | 5,063 |
| *Non-penicillin β-lactams (J01D)* | 218 | 181 | 37 | 57 | 275 | 296 | 233 | 63 | 206 | 502 |
| *Sulphonamides and trimethoprim (J01E)* | 356 | 286 | 70 | 167 | 523 | 647 | 530 | 117 | 526 | 1,173 |
| *Macrolides, lincosamides and streptogramins (J01F)* | 430 | 336 | 94 | 105 | 535 | 1,195 | 944 | 251 | 340 | 1,535 |
| *Aminoglycosides (J01G)* | 0 | 0 | 0 | 0 | 0 | 1 | 1 | 0 | 0 | 1 |
| *Quinolones (J01M)* | 591 | 497 | 94 | 167 | 758 | 1,251 | 1,027 | 224 | 735 | 1,986 |
| *Other antibacterials (J01X and P01AB)* | 930 | 718 | 212 | 461 | 1,391 | 1,089 | 880 | 209 | 1,003 | 2,092 |

**Table S3. Effect of proton pump inhibitors (PPI) and different classes of antibiotics on the risk of *Clostridioides difficile* infection (CDI) and recurrence, expressed as odds ratios and 95% confidence intervals**

|  | **First episode (compared to controls)** | | **Recurrence (compared to no recurrence)** | |
| --- | --- | --- | --- | --- |
|  | **Recent**  **(0 – 30 days)** | **Preceding 5 months**  **(31 – 180 days)** | **Recent**  **(0 – 30 days)** | **Preceding 5 months**  **(31 – 180 days)** |
| **Model with all antibiotics combined** |  |  |  |  |
| Proton pump inhibitors (PPI) | 2.65 [2.54; 2.76]* | 2.08 [2.01; 2.15]* | 1.03 [0.94; 1.12] | 1.12 [1.03; 1.21]* |
| Systemic antibiotics (AB) | 15.37 [14.83; 15.93]* | 5.42 [5.26; 5.57]* | 1.30 [1.23; 1.38]* | 1.23 [1.16; 1.31]* |
| Interaction term PPI and AB | 0.43 [0.39; 0.47]# | 0.81 [0.77; 0.85]# | 0.98 [0.86; 1.11] | 0.92 [0.82; 1.02] |
| **Model with antibiotic subtypes** |  |  |  |  |
| Proton pump inhibitors (PPI) | 2.62 [2.51; 2.73]* | 2.14 [2.07; 2.22]* | 1.04 [0.96; 1.13] | 1.09 [1.02; 1.18]* |
| Systemic antibiotics (AB) |  |  |  |  |
| *Tetracyclines (J01A)* | 1.51 [1.27; 1.78]* | 1.49 [1.38; 1.62]* | 0.64 [0.43; 0.91]* | 0.90 [0.76; 1.06] |
| *Penicillins (J01C)* | 7.67 [7.28; 8.10]* | 2.53 [2.44; 2.63]* | 1.08 [0.99; 1.17] | 1.08 [1.00; 1.16]* |
| *Non-penicillin β-lactams (J01D)* | 13.25 [11.23; 15.62]* | 3.09 [2.76; 3.47]* | 1.28 [1.07; 1.53]* | 0.99 [0.82; 1.19] |
| *Sulphonamides and trimethoprim (J01E)* | 6.19 [5.46; 7.03]* | 2.12 [1.95; 2.30]* | 0.97 [0.82; 1.16] | 1.07 [0.92; 1.24] |
| *Macrolides, lincosamides and streptogramins (J01F)* | 26.47 [23.65; 29.63]* | 14.81 [13.91; 15.77]* | 1.42 [1.26; 1.58]* | 1.38 [1.26; 1.50]* |
| *Aminoglycosides (J01G)* | 1.31 [0.27; 6.22] | 0.84 [0.20; 3.49] | 0.65 [0.03; 3.67] | 0.61 [0.03; 3.39] |
| *Quinolones (J01M)* | 8.99 [8.18; 9.87]* | 3.34 [3.14; 3.55]* | 1.11 [1.00; 1.24] | 1.04 [0.93; 1.15] |
| *Other antibacterials incl. metronidazole (J01X and P01AB)* | 13.73 [12.78; 14.75]* | 2.82 [2.65; 2.99]* | 1.43 [1.31; 1.56]* | 1.03 [0.92; 1.14] |
| *Interaction term PPI and Tetracyclines (J01A)* | 0.58 [0.39; 0.85]# | 0.82 [0.72; 0.93]# | 1.75 [0.84; 3.52] | 1.16 [0.91; 1.47] |
| *Interaction term PPI and Penicillins (J01C)* | 0.58 [0.51; 0.66]# | 0.87 [0.82; 0.93]# | 0.98 [0.82; 1.17] | 0.93 [0.83; 1.04] |
| *Interaction term PPI and Non-penicillin β-lactams (J01D)* | 0.43 [0.29; 0.62]# | 0.68 [0.56; 0.83]# | 0.70 [0.46; 1.03] | 1.09 [0.82; 1.44] |
| *Interaction term PPI and Sulphonamides and trimethoprim (J01E)* | 0.67 [0.52; 0.87]# | 1.14 [1.00; 1.30]# | 1.20 [0.86; 1.64] | 1.00 [0.81; 1.22] |
| *Interaction term PPI and Macrolides, lincosamides and streptogramins (J01F)* | 0.30 [0.23; 0.40]# | 0.48 [0.43; 0.53]# | 0.96 [0.74; 1.24] | 0.95 [0.83; 1.09] |
| *Interaction term PPI and Aminoglycosides (J01G)* | - | - | - | 1.29 [0.10; 31.08] |
| *Interaction term PPI and Quinolones (J01M)* | 0.75 [0.60; 0.94]# | 0.88 [0.80; 0.98]# | 0.75 [0.58; 0.96]# | 0.95 [0.81; 1.11] |
| *Interaction term PPI and Other antibacterials (J01X/P01AB)* | 0.30 [0.26; 0.35]# | 0.65 [0.58; 0.71]# | 1.03 [0.85; 1.24] | 1.09 [0.93; 1.28] |

**Table S4. Predictors for recurrence of *Clostridioides difficile* infection (CDI) using those with a single episode as reference; by period of exposure to antibiotics and proton pump inhibitors.**

|  | **First episode (compared to controls)** | | **Recurrence (compared to no recurrence)** | |
| --- | --- | --- | --- | --- |
|  | **Recent**  **(0 – 30 days)** | **Preceding 5 months (31 – 180 days)** | **Recent (0 – 30 days)** | **Preceding 5 months**  **(31 – 180 days)** |
| Female sex | - | - | 1.14 [1.08; 1.20] | 1.14 [1.08; 1.20] |
| Age at first diagnosis, per 10 years | - | - | 1.04 [0.99; 1.02] | 1.02 [0.99; 1.02] |
| Region of birth, non-Nordic | 0.80 [0.76; 1.31] | 0.77 [0.73; 0.81] | 0.92 [0.82; 1.04] | 0.92 [0.82; 1.04] |
| Charlson comorbidity score, per point increase | 1.30 [1.29; 1.31] | 1.28 [1.27; 1.29] | 1.03 [1.02; 1.04] | 1.03 [1.01; 1.04] |
| Inflammatory bowel disease | 4.84 [4.50; 5.19] | 4.53 [4.23; 4.85] | 1.20 [1.07; 1.34] | 1.19 [1.06; 1.33] |
| Haematological diseases | 3.10 [3.02; 3.19] | 2.88 [2.80; 2.95] | 1.06 [1.01; 1.12] | 1.05 [0.99; 1.10] |
| History of CDI | - | - | 1.84 [1.50; 2.23] | 1.81 [1.48 2.20] |
| Aspirin | 0.86 [0.83; 0.90] | 0.96 [0.93; 0.99] | 0.95 [0.87; 1.02] | 1.00 [0.93; 1.06] |
| H2-receptor antagonists | 1.72 [1.41; 2.11] | 1.60 [1.42; 1.80] | 1.04 [0.71; 1.47] | 1.06 [0.84; 1.32] |
| Non-steroidal anti-inflammatory drugs (NSAIDs) | 1.24 [1.16; 1.33] | 1.07 [1.03; 1.11] | 1.01 [0.88; 1.15] | 0.97 [0.89; 1.05] |
| Proton pump inhibitors (PPI) | 2.65 [2.54; 2.76] | 2.08 [2.00; 2.15] | 1.03 [0.94; 1.12] | 1.12 [1.03; 1.21] |
| Antibiotics (AB) | 15.37 [14.83; 15.93] | 5.41 [5.26; 5.57] | 1.30 [1.23; 1.38] | 1.23 [1.16; 1.31] |
| Interaction PPI and AB | 0.43 [0.39; 0.47] | 0.81 [0.77; 0.85] | 0.98 [0.86; 1.11] | 0.92 [0.82; 1.02] |

All models were adjusted for region of birth, chronic comorbidity score (continuous variable), inflammatory bowel disease, haematological diseases, aspirin, NSAIDs use, and H2-receptor antagonists use. The recurrence model was additionally adjusted for sex and age (continuously) which were used for matching and could therefore not be assessed in the CDI risk model.

**References**

1. Wettermark B, Hammar N, Fored CM *et al.* The new Swedish Prescribed Drug Register--opportunities for pharmacoepidemiological research and experience from the first six months. *Pharmacoepidemiology and drug safety* 2007; **16**: 726-35.

2. Ludvigsson JF, Andersson E, Ekbom A *et al.* External review and validation of the Swedish national inpatient register. *BMC Public Health* 2011; **11**: 450.

3. Barlow L, Westergren K, Holmberg L *et al.* The completeness of the Swedish Cancer Register: a sample survey for year 1998. *Acta Oncol* 2009; **48**: 27-33.

4. Brusselaers N. Prescribed Drugs and the Microbiome. *Gastroenterol Clin North Am* 2019; **48**: 331-42.

5. Brusselaers N, Lagergren J. The Charlson Comorbidity Index in Registry-based Research. *Methods of information in medicine* 2017; **56**: 401-6.

6. Ludvigsson JF, Appelros P, Askling J *et al.* Adaptation of the Charlson Comorbidity Index for Register-Based Research in Sweden. *Clin Epidemiol* 2021; **13**: 21-41.

7. Huang J, Kockum I, Stridh P. Interaction between two exposures: determining odds ratios and confidence intervals for risk estimates. *arXiv* 2020; **arXiv:2008.01479**.
